# Supplementary material for: Amplification of heat extremes by plant CO2 physiological forcing
Source: Nat Commun. 2018 Mar 15;9:1094. doi: 10.1038/s41467-018-03472-w (PMC5854667; doi:10.1038/s41467-018-03472-w)
Supplement: Supplementary file 1 — Supplementary Information [file 41467_2018_3472_MOESM1_ESM.pdf]

Supplementary Information for

Amplification of heat extremes by plant CO_2 physiological forcing

Skinner et al.

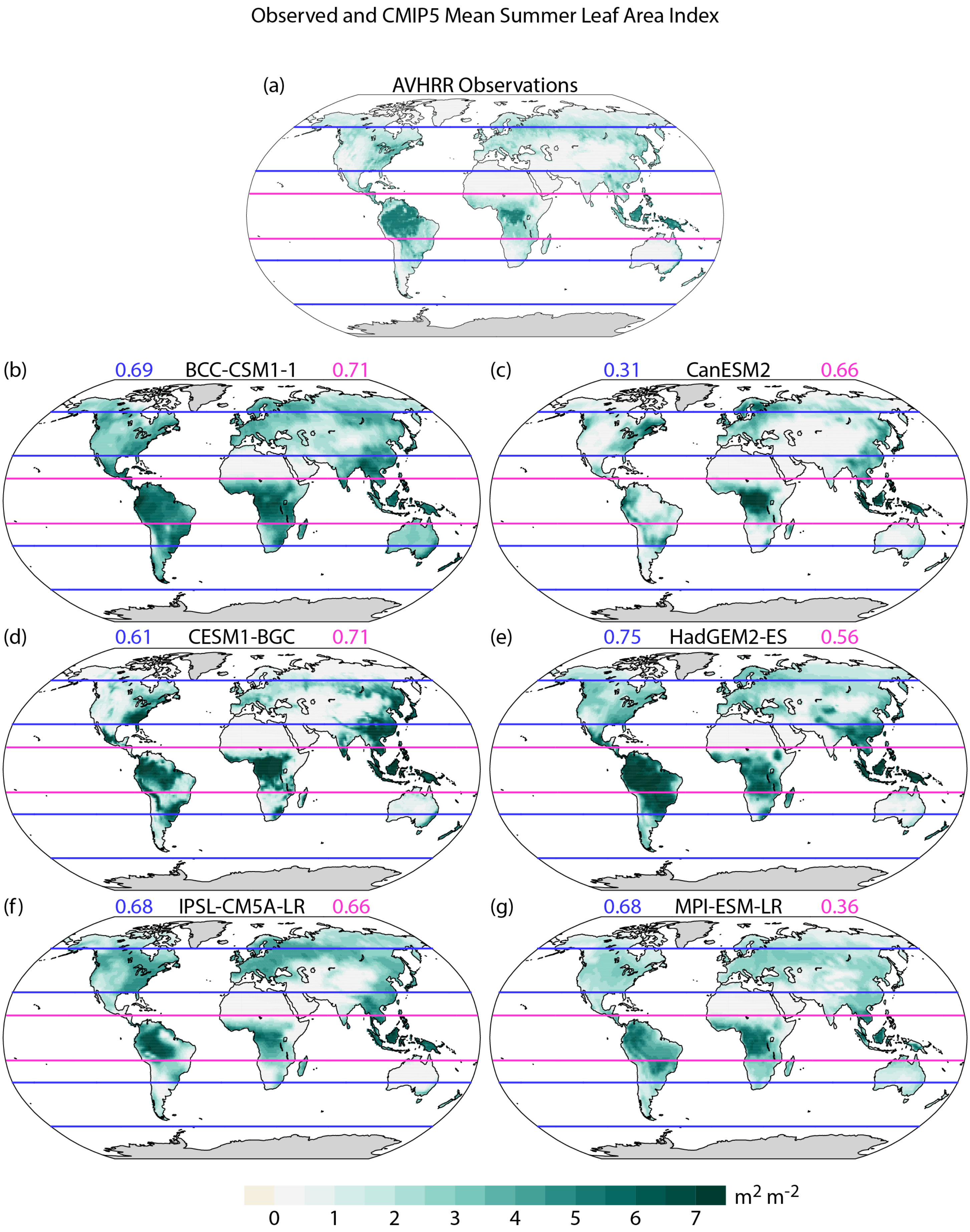

Supplementary Figure 1. Comparison of CMIP5 and satellite-based leaf area index (LAI). Mean summer LAI from (a) the AVH15C1 satellite-derived observation dataset (years 1990 - 2010), and (b-g) the *TotalCO2* simulations from CMIP5 (years 22-32, CO2 values of ~ 355 - 390 ppm). Model years 22 - 32 are chosen to closely match the CO2 values during the years 1990 - 2010. Centered pattern correlation values between AVH15C1 and model data for the tropics (15°S - 15°N, pink) and mid-latitudes (30°N/S - 60°N/S, blue) are listed above each panel. JJAS (DJFM) values are used in the Northern (Southern) Hemisphere.

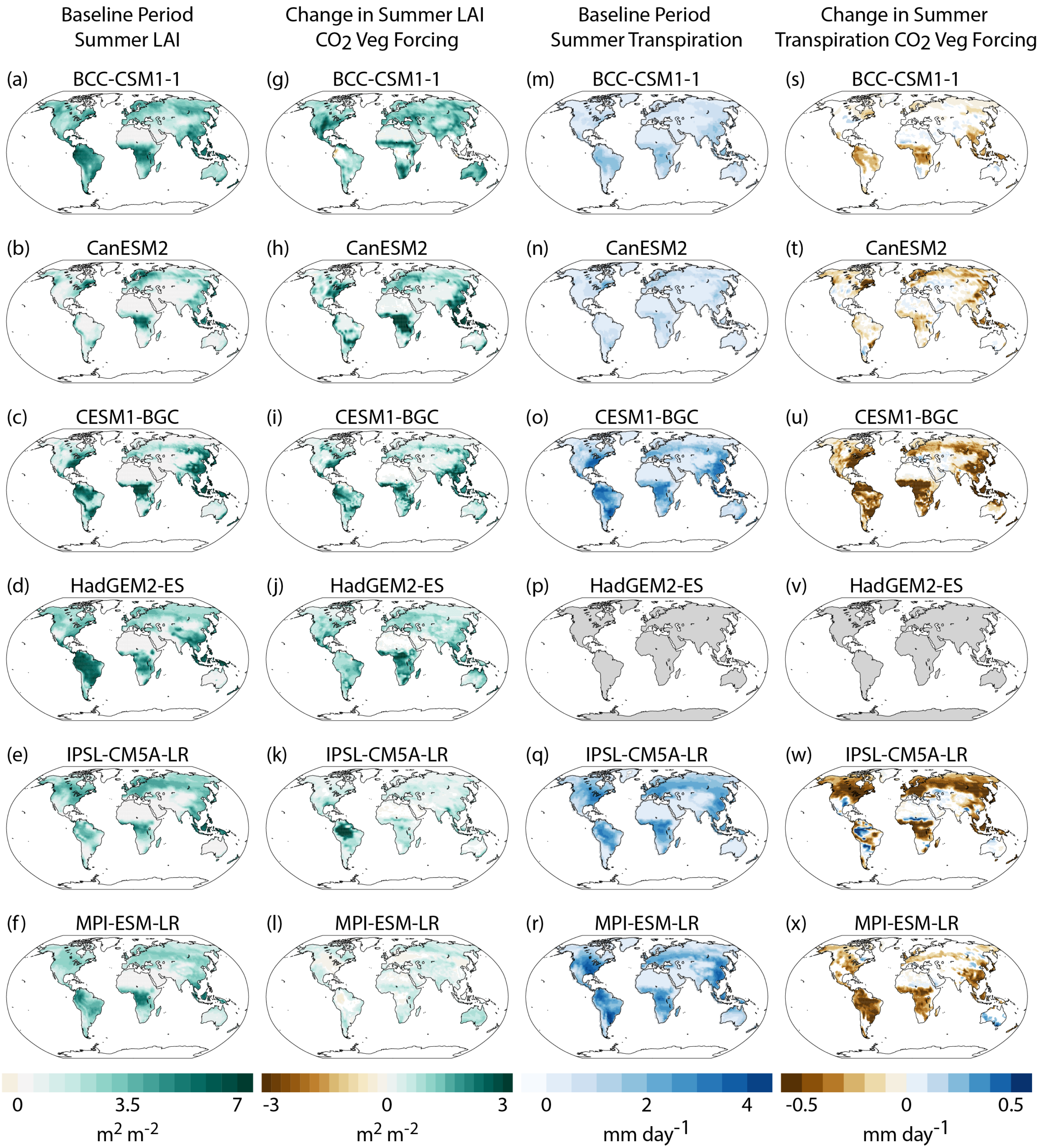

Supplementary Figure 2. Simulated baseline period and statistically significant change in LAI and transpiration. Mean summer (a-f) reference climate LAI (final 30 years of the *RadCO2* simulation), (g-l) change in LAI from CO2 vegetaton forcing, (m-r) refrence climate transpiration, and (s-x) change in transpiration from CO2 vegetation forcing. JJAS (DJFM) values are used in the Northern (Southern) Hemisphere. HadGEM2-ES does not output a transpiration variable.

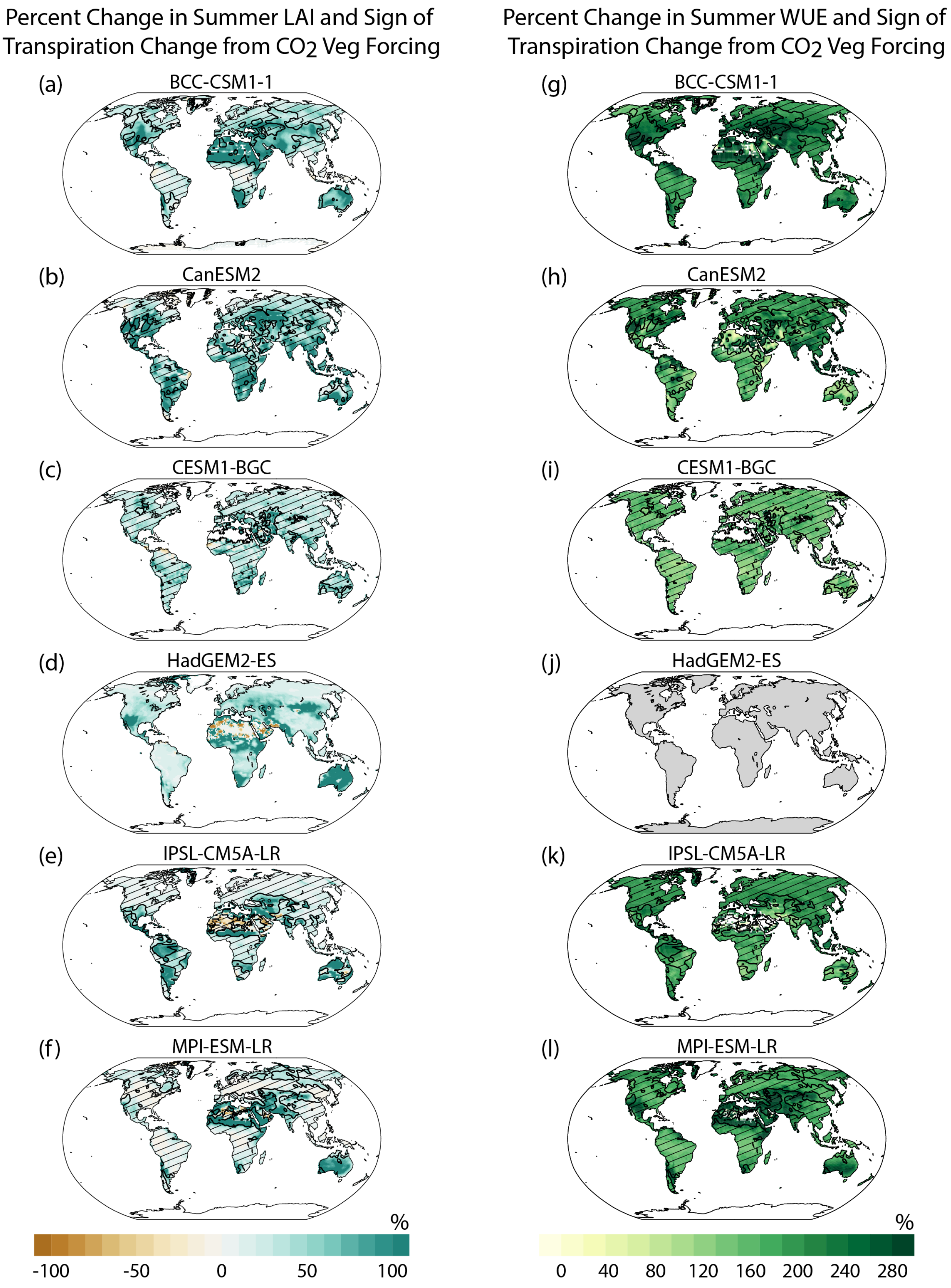

Supplementary Figure 3. Percent change in LAI and WUE from CO2 vegetation forcing. Percent change in mean summer (a-f) LAI and (g-l) WUE from CO2 vegetation forcing (shading). Dashed lines indicate where the sign of mean summer transpiration change from CO2 vegetation forcing is negative. The maximum and minimum percent LAI changes are capped at +100% and -100%, respectively, in (a-f). JJAS (DJFM) values are used in the Northern (Southern) Hemisphere in all panels. HadGEM2-ES does not output transpiration and therefore is not used to analyze WUE (defined here as the ratio of gross primary productivity to transpiration).

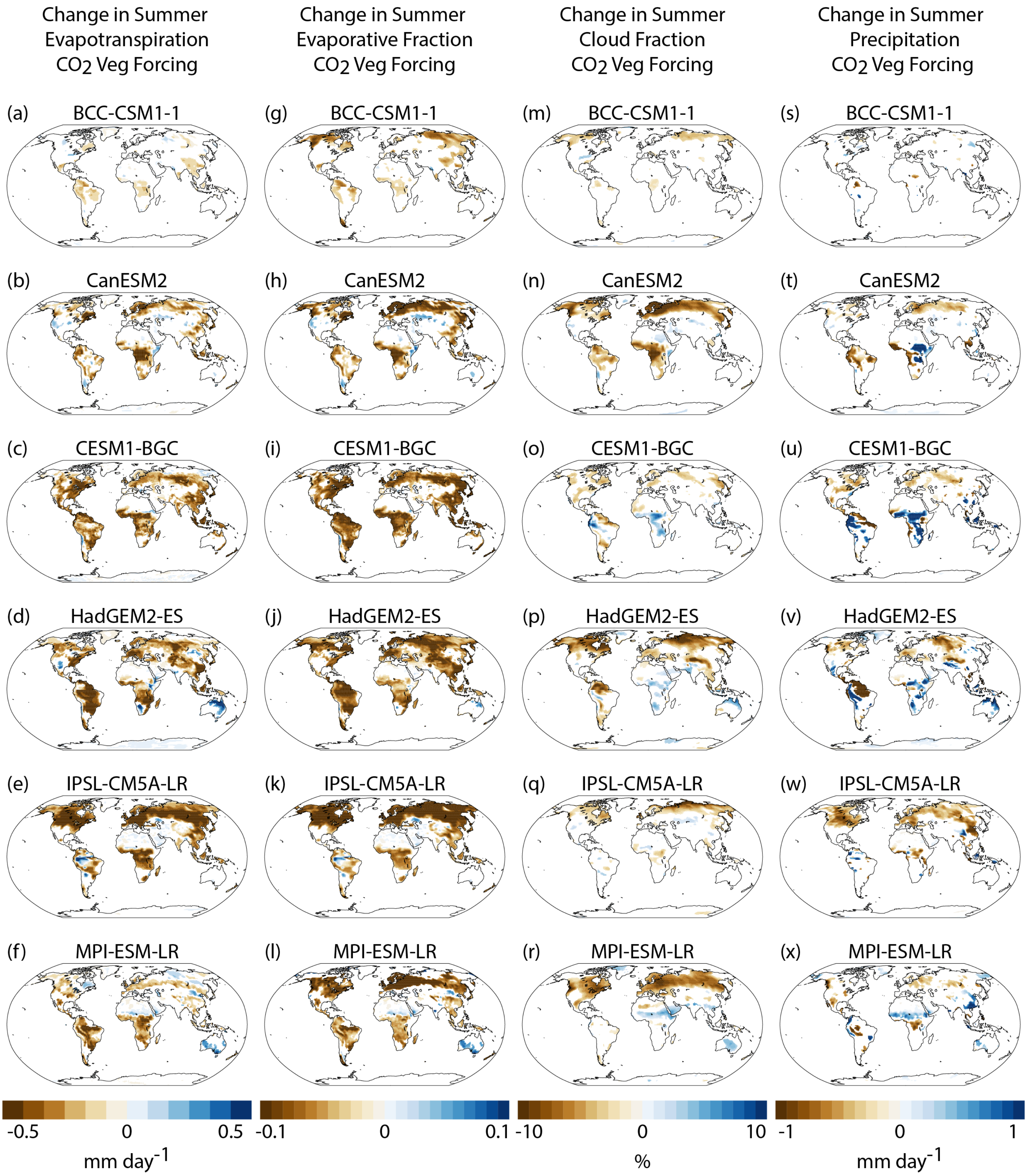

Supplementary Figure 4. Statistically significant changes in surface hydrological variables, clouds, and precipitation from CO2 vegetation forcing over land. Change in mean summer (a-f) evapotranspiration, (g-l) evaporative fraction, (m-r) total cloud fraction, and (s-x) precipitation from CO2 vegetaton forcing. JJAS (DJFM) values are used in the Northern (Southern) Hemisphere.

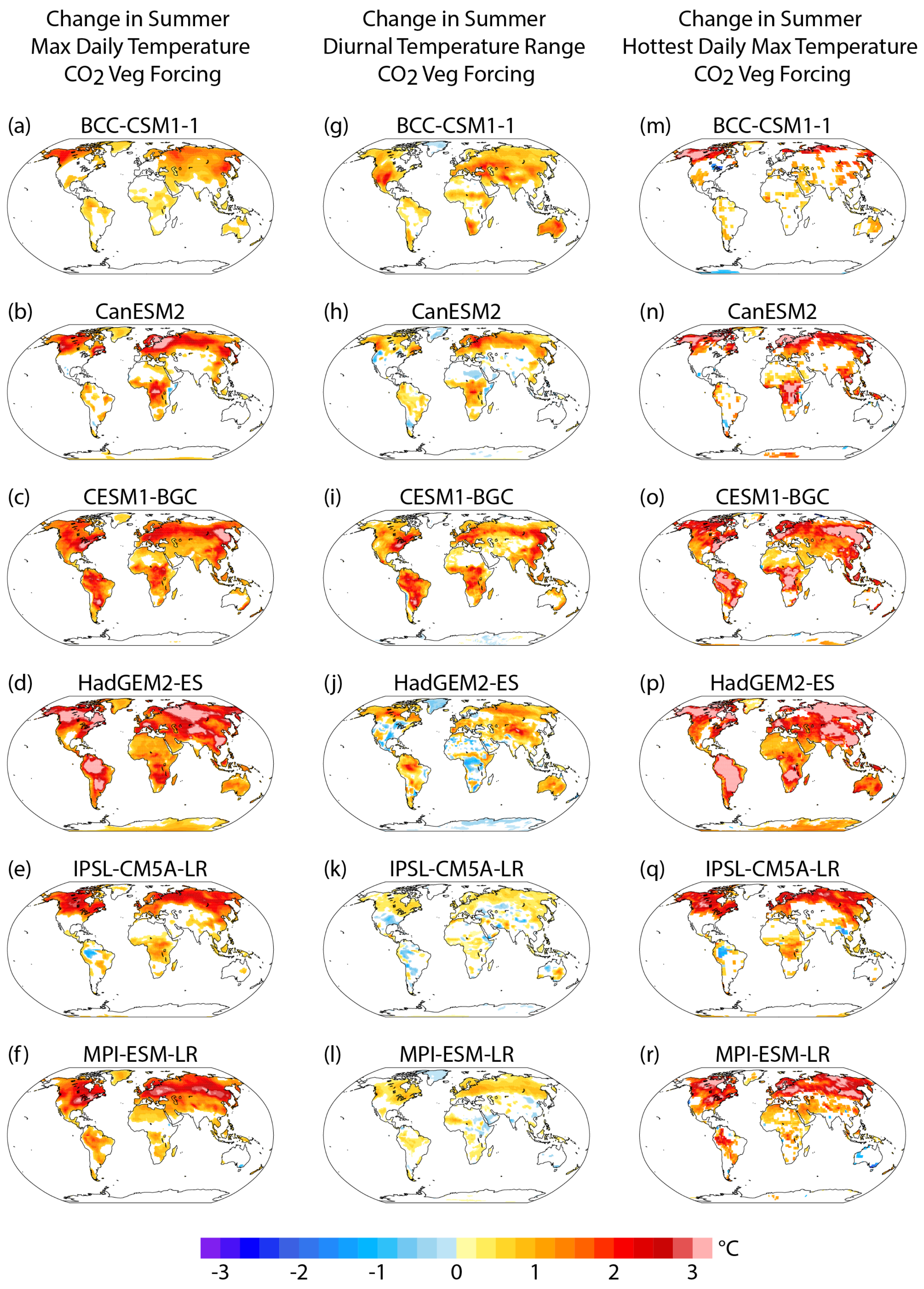

Supplementary Figure 5. Statistically significant changes in near-surface temperature from CO2 vegetation forcing over land. Change in mean summer (a-f) maximum daily 2-meter temperature, (g-l) diurnal 2-meter temperature range, and (m-r) hottest daily maximum 2-meter temperature from CO2 vegetaton forcing. JJAS (DJFM) values are used in the Northern (Southern) Hemisphere.

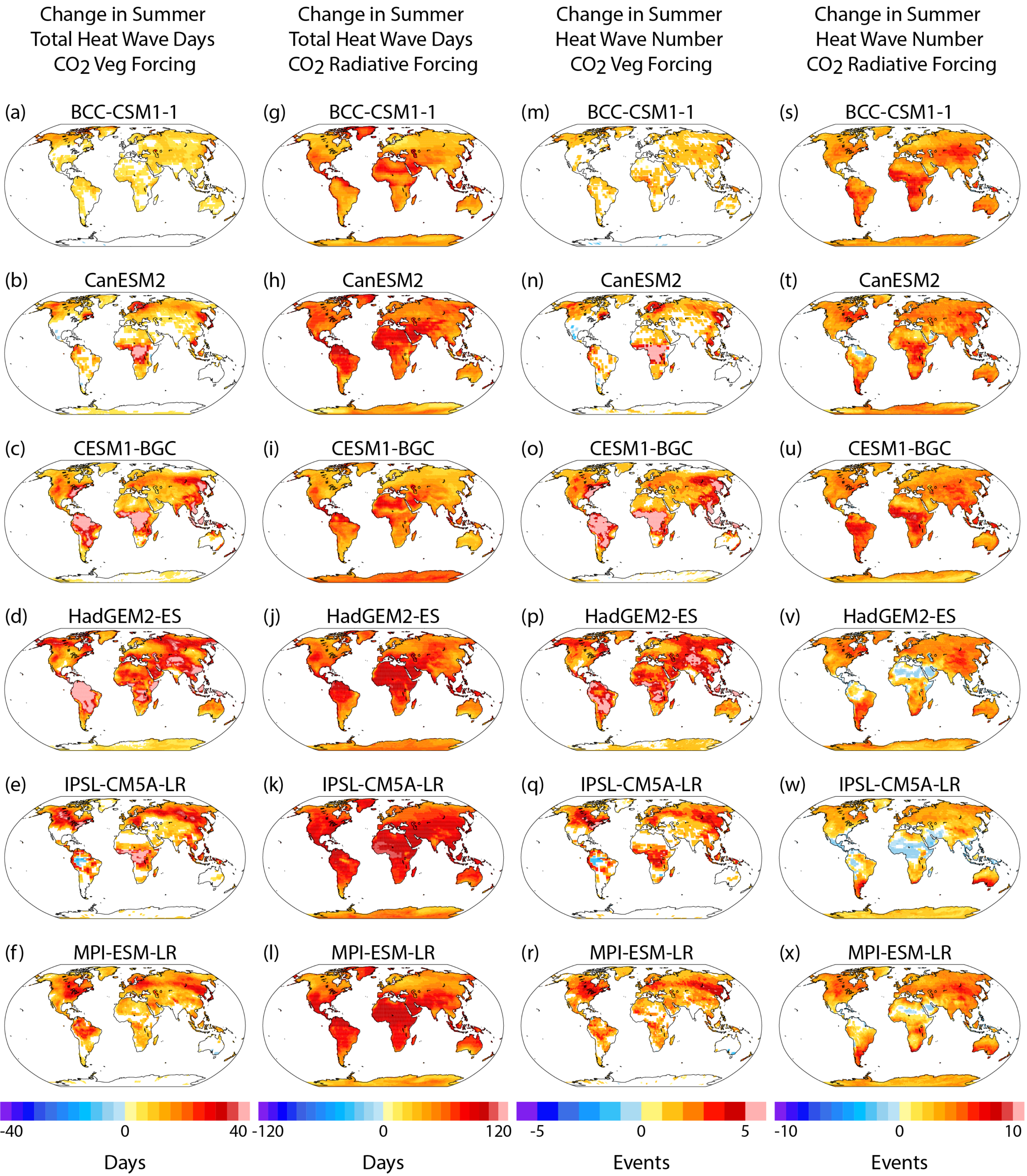

Supplementary Figure 6. Statistically significant changes in heat wave metrics from CO2 forcing over land. Change in mean summer total heat wave days (HWTD) from (a-f) CO2 vegetation forcing, and (g-l) CO2 radiative forcing . Change in mean summer heat wave number (HWN) from (m-r) CO2 vegetation forcing, and (s-x) CO2 radiative forcing. See Methods for heat wave metric definitions. JJAS (DJFM) values are used in the Northern (Southern) Hemisphere. Note the different scales for vegetation and radiative forcing.

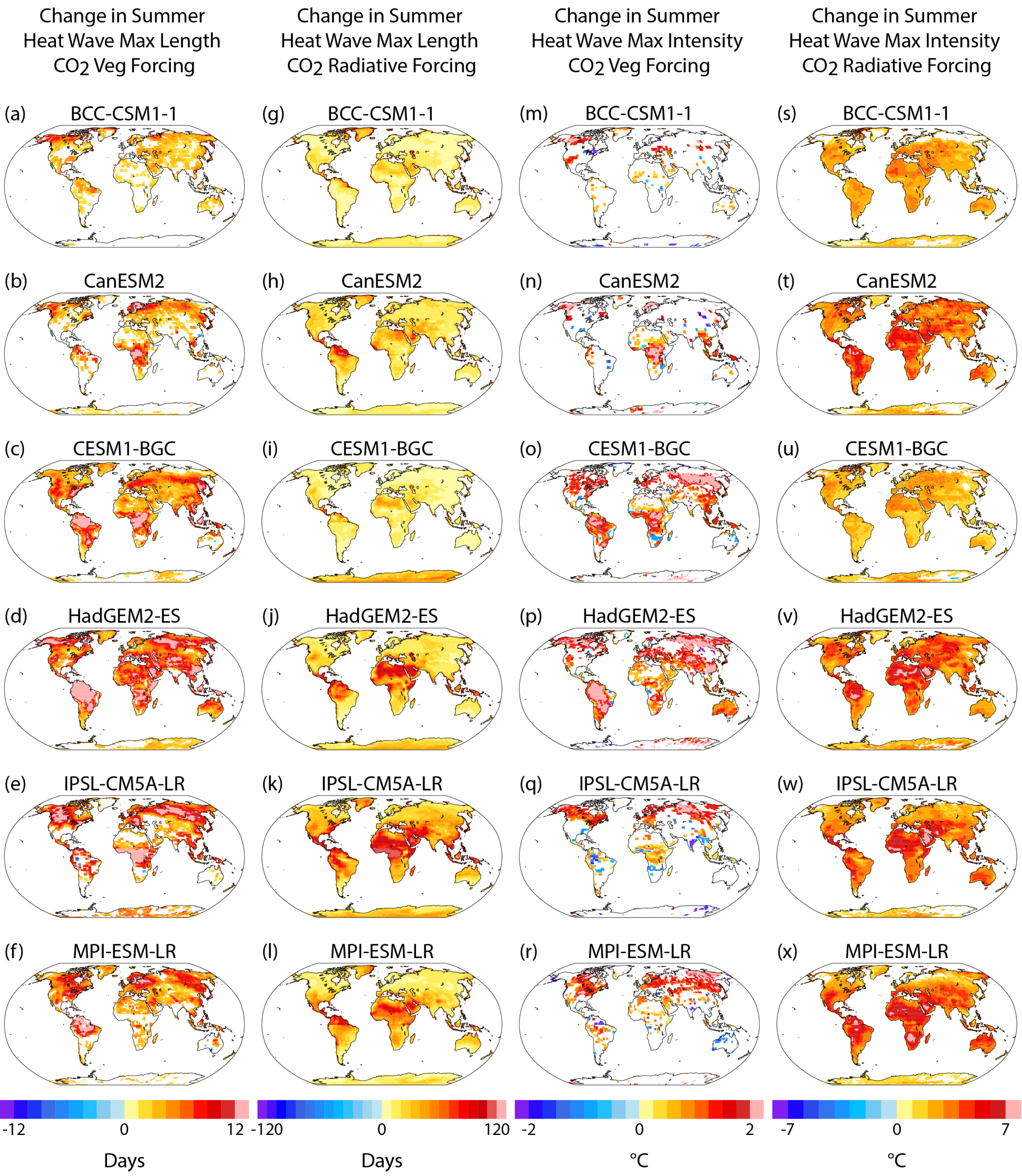

Supplementary Figure 7. Statistically significant changes in heat wave metrics from CO2 forcing over land. Change in mean summer maximum heat wave length (HWML) from (a-f) CO2 vegetation forcing, and (g-l) CO2 radiative forcing. Change in mean summer heat wave maximum intensity (HWMI) from (m-r) CO2 vegetation forcing, and (s-x) CO2 radiative forcing. See Methods for heat wave metric definitions. JJAS (DJFM) values are used in the Northern (Southern) Hemisphere. Note the different scales for vegetation and radiative forcing.

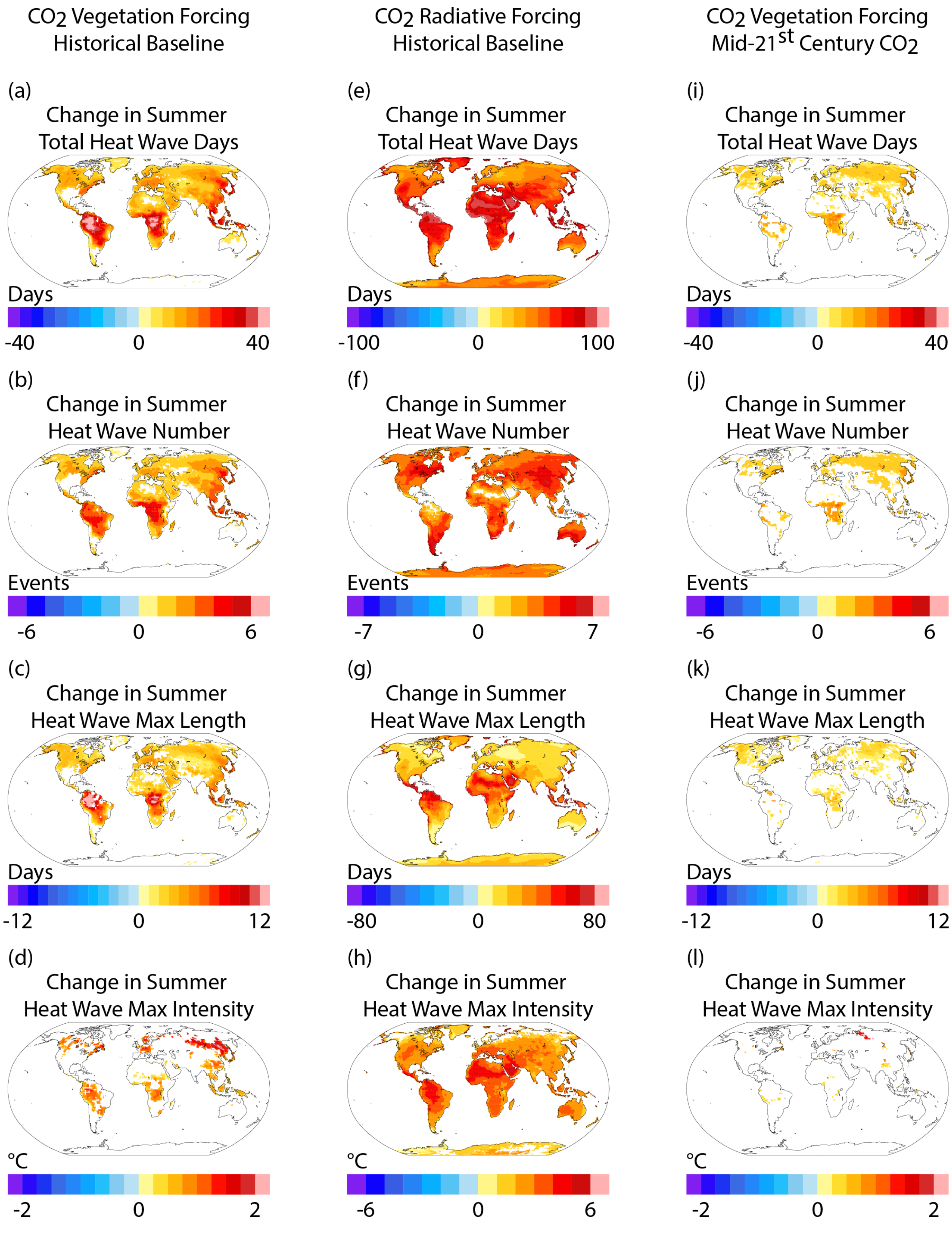

Supplementary Figure 8. Statistically significant changes in heat wave metrics from CO2 forcing over land. Multi-model mean change in summer (a, e, i) total heat wave days (HWTD), (b, f, j) heat wave number (HWN), (c, g, k) heat wave maximum length (HWML), and (d, h, l) heat wave maximum intensity (HWMI). The changes are in response to (a-d) CO2 vegetation forcing using a historical reference climate (final 30 years from VegCO2 minus first 30 years from VegCO2), (e-h) CO2 radiative forcing using a historical reference climate (final 30 years from RadCO2 minus first 30 years from RadCO2), and (i-l) CO2 vegetation forcing for a mid-21st century CO2 concentration (years 58-87 from TotalCO2 minus years 58-87 from RadCO2). See Methods for heat wave metric definitions. JJAS (DJFM) values are used in the Northern (Southern) Hemisphere. Note the different scales for vegetation and radiative forcing.

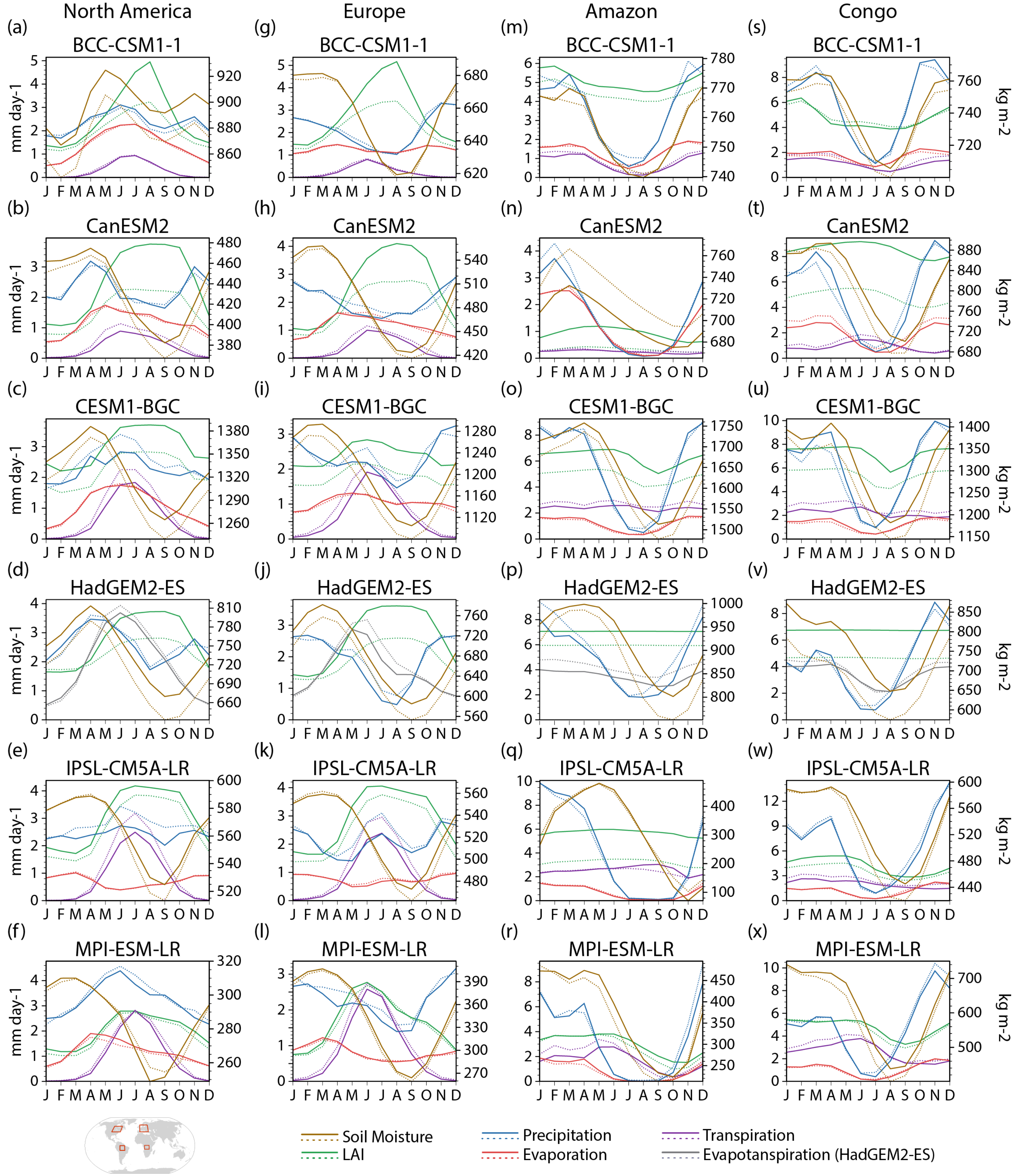

Supplementary Figure 9. Annual cycle of vegetation and hydrologic variables in simulations with and without future CO2 vegetation forcing. CMIP5 multi-model mean area-weighted averages of total column soil moisture (brown), leaf area index (LAI) (green), precipitation (blue), evaporation (red), transpiration (purple), and evapotranspiration (grey) for portions of (a-f) North America (40°N-55°N, 100°W-70°W), (g-l) Europe (40°N-60°N, 0°-30°E), (m-r) the Amazon basin (0°-15°S, 65°W-50°W) and (s-x) the Congo basin (0°-10°S, 15°E-30°E). Dashed (solid) lines represent the final 30 years of *RadCO2* (*TotalCO2*). LAI (unitless), precipitation, evaporation, transpiration, and evapotranspiration correspond to the left y-axis. Soil moisture corresponds to the right y-axis. Evapotranspiration is shown for HadGEM2-ES.

Supplementary Tables

Supplementary Table 1. CMIP5 model resolution and land surface components

Model Name	Atmosphere Model Resolution	Dynamic Vegetation	Stomatal Conductance Scheme	Soil Depth, Number of Layers
BCC-CSM1-1	T42L26 ~ 2.8°	No	Ball-Berry	3.43m, 10 layers
CanESM2	T63L35 ~ 2.8°	No	Leuning	4.10m, 3 layers
CESM1-BGC	0.9° x 1.25°	No	Ball-Berry	3.80m, 10 layers
HadGEM2-ES	1.25° x 1.875°	Yes	Simplified Leuning	3.00m, 4 layers
IPSL-CM5A-LR	1.9° x 3.75°	No	Ball-Berry	Varies, 2 layers
MPI-ESM-LR	T63L47 ~ 1.875°	Yes	Knorr	Varies, 1 layer

Only hydrologically-active soil depths and layers are listed. IPSL-CM5A-LR and MPI-ESM-LR utilize bucket models with geographically varying soil depths depending on root distribution.

Supplementary Table 2. Average rate of LAI change and transpiration change during the first and second doubling of CO2

Model Name	Δ LAI 284–568 ppm ($m^2 m^{-2} ppm^{-1}$)	Δ LAI 568–1132 ppm ($m^2 m^{-2} ppm^{-1}$)	Δ Transp 284–568 ppm ($mm\ ppm^{-1}$)	Δ Transp 568–1132 ppm ($mm\ ppm^{-1}$)
**Tropics (15°S–15°N)**				
BCC-CSM1-1	1.49^{-3}	0.789^{-3}	-3.81^{-4}	-1.62^{-4}
CanESM2	3.29^{-3}	1.50^{-3}	-1.72^{-4}	-1.53^{-4}
CESM1-BGC	2.24^{-3}	1.94^{-3}	-7.65^{-4}	-5.66^{-4}
HadGEM2-ES	2.48^{-3}	1.45^{-3}	-2.97^{-4}	-6.00^{-4}
IPSL-CM5A-LR	1.71^{-3}	0.864^{-3}	-3.38^{-4}	-2.76^{-4}
MPI-ESM-LR	0.424^{-3}	0.119^{-3}	-4.34^{-4}	-3.69^{-4}
**Subtropics (15°S/N–30°S/N)**				
BCC-CSM1-1	2.76^{-3}	0.746^{-3}	$+0.486^{-4}$	-0.824^{-4}
CanESM2	1.29^{-3}	0.752^{-3}	-0.154^{-4}	-0.542^{-4}
CESM1-BGC	1.31^{-3}	1.13^{-3}	-3.72^{-4}	-1.83^{-4}
HadGEM2-ES	1.88^{-3}	0.848^{-3}	$+2.91^{-4}$	-1.44^{-4}
IPSL-CM5A-LR	0.507^{-3}	0.291^{-3}	-2.36^{-4}	-0.426^{-4}
MPI-ESM-LR	0.542^{-3}	0.236^{-3}	-3.34^{-4}	-3.20^{-4}
**Extratropics (30°S/N–70°S/N)**				
BCC-CSM1-1	2.25^{-3}	0.805^{-3}	$+0.312^{-4}$	-0.604^{-4}
CanESM2	1.06^{-3}	0.822^{-3}	-0.622^{-4}	-0.578^{-4}
CESM1-BGC	0.803^{-3}	0.630^{-3}	-2.67^{-4}	-2.21^{-4}
HadGEM2-ES	1.79^{3}	0.686^{-3}	-0.451^{-4}	-1.72^{-4}
IPSL-CM5A-LR	0.534^{-3}	0.152^{-3}	-3.02^{-4}	-2.50^{-4}
MPI-ESM-LR	0.774^{-3}	0.293^{-3}	$+0.514^{-4}$	-0.744^{-4}

Rates of change expressed as: ($\Delta LAI/\Delta CO_2$ ppm) and ($\Delta Tran/\Delta CO_2$ ppm).

Supplementary Note 1

Earth system models (ESMs) exhibit varying skill in simulating global spatial patterns of summer LAI (Supplementary Figure 1) (1). In the mid-latitudes, most models correctly simulate greatest LAI in the deciduous broadleaf forests of the eastern United States and Europe, and in the needleleaf forests of Canada, northern Europe and Asia (mid-latitude centered spatial correlations with AVH15C1 range from 0.31 to 0.75). MPI-ESM-LR (correlation of 0.31) largely misses this pattern. Similarly, most models correctly simulate high LAI in the tropical evergreen broadleaf forests of the Amazon, Congo, and Maritime Continent (tropical, centered pattern correlations with AVH15C1 range from 0.36 to 0.71), though a dry bias in CanESM2 (2) contributes to substantial underestimation of Amazon LAI (correlation of 0.36). Some of the regional-scale LAI differences between AVH15C1 and the models may be driven by a lack of prescribed land use change in several of the ESMs. A detailed evaluation of simulated LAI in the CMIP5 ensemble can be found in (1).

Supplementary References

1 Mahowald, N. *et al.* Projections of leaf area index in earth system models. *Earth Syst. Dynam.* **7**, 211-229 (2016).

2 Ahlström, A. *et al.* Hydrologic resilience and Amazon productivity. *Nature Communications* **8**, 387, doi:10.1038/s41467-017-00306-z (2017).
